# Supplementary material for: Computational screening of potential glioma-related genes and drugs based on analysis of GEO dataset and text mining
Source: PLoS One. 2021 Feb 26;16(2):e0247612. doi: 10.1371/journal.pone.0247612 (PMC7909668; doi:10.1371/journal.pone.0247612)
Supplement: Attachment — Submitted filename: Response to Reviewers.docx [file pone.0247612.s001.docx]

Dear Editor Edwin Wang and Reviewers:

We are very grateful to Reviewer for reviewing the paper so carefully. We have carefully considered the suggestion of Reviewer and make some changes.

Responds to the Editor Wang’s comments:

1 Please clarify in the GO/KEGG analysis the p values were FDR-adjusted.

Answer: We appreciate and thank for the detailed Editor Wang of our manuscript. We are very sorry for our negligence of the explanation and we have adjusted the P values by FDR in GO and KEGG analyses.

2 Please clarify if statistical measures have been used in the drug-gene analysis.

Answer: The source of the drug score in the drug-gene database is based on its source. For example, one score is derived from published articles while another score is derived from the database. This is a descriptive conclusion without using statistical method. Thank you very much for your great efforts on our manuscript.

Responds to the reviewers' comments:

Reviewer #1：

1. There are several glioma gene expression datasets in GEO database. Why did the authors consider only GSE31095, which is a relatively old, small-size microarray dataset in the analysis? Considering consensus DEGs in multiple gene expression datasets will prominently improve the confidence of the resulted gene list.

Answer: We appreciate it very much for this good suggestion. However, this data set has been already used by our research team in relevant exploration and mining, so I further analyzed and mined it with text mining through generating letters. Coincidentally, I got several genes that I was studying, such as RPL8 and RPSA.

2. As for the drug analysis, the authors picked the drugs targeting hub genes (which were mostly known cancer driver genes) as the potential glioma-related drugs. The authors can implement gene expression-based drug analysis like Connectivity Map analysis to validate the predicted drugs.

Answer: We would like to thank the reviewer fort his comment. As reviewers have pointed out, the drugs selected are indeed those with known oncogenes, but they are not included in the treatment guidelines for gliomas. Therefore, in this study, we screened out these potential tumor drugs to lay a foundation for the following basic experiments, hoping to expand the indications of these drug therapy and provide new possibilities for the targeted therapy of glioma in the future.

3. The threshold of statistical significance was vague: P-value, adjusted P-value, or FDR, which one was used should be consistent.

Answer: We are very sorry for our negligence of the explanation. We have already adjusted the statistical thresholds to FDR/ ad-value (tip: FDR and ad-value are the same thing). We would like to thank the reviewer also fort his comment.

4. Grammatical errors, awkward expressions or typos could be observed in nearly everyy paragraph of the manuscript. For example, the title should be “Computational Screening of Potential Glioma-Related Genes and Drugs Based on Analysis of GEO Dataset and Text Mining” rather than “Identification of Glioma-Related Potential Genes and Drugs: Based on GEO Database and Mining text”. Careful language editing by a native speaker is necessary before further consideration of the manuscript.

Answer: We apologize for the poor language of our manuscript. The manuscript has been revised by a native English speaker for language corrections.  We really hope that the flow and language level have been substantially improved. Many thanks go to reviewers, and we are feel so warm for your suggestions.

Reviewer #2：

1. Please clearly state the major innovation of this work.

Answer: We thank the reviewer. The innovation of our research lies in the cross-combination of data sets in GEO database and text mining. Through the bioinformatics analysis, differential genes are screened out and potential targeted drugs are further explored through differential genes, which will provide new targets and indications for the clinical treatment of glioma.

2. I want to know whether the researches for verifying your identification (the researches of Swoboda et al. and Shi et al.) are included in the GenCLIP3 platform for text mining.

Answer: Dear reviewer, I think you may have misunderstood. The research of Swoboda et al. and Shi et al. is only used to prove that the differentially expressed genes I have obtained are related to other tumors, and these differentially expressed genes are derived from the GenCLIP3 platform and the GSE31095 data set. Therefore, only differentially expressed genes are included in the GenCLIP3 platform. We appreciate and thank for the detailed review of our manuscript.

3. I want to know whether this method can also be used for the analysis of other cancer.

Answer: We would like to thank the reviewer fort his comment. I think it can be used for analyzing many cancers. Because bioscientific research is based on big data survey research, this also means that as long as there is reliable and sufficient tumor sample data, it can be fully applied to other research. Therefore, our current glioma research is not a special case, but a microcosm of research directions.

4. You should revise your English writing carefully and eliminate small errors in the paper to make the paper easier to understand.

Answer: We apologize for the poor language of our manuscript. The manuscript has been revised by a native English speaker for language corrections. We really hope that the flow and language level have been substantially improved. We would like to thank the reviewer also fort his comment.

5. Could you give some discussions whether your method could be used to predict glioma-related potential non-coding RNAs as the future direction of this work (PMIDs: 29939227, 29045685, 30142158, and 27345524)?

Answer: Thank you for your valuable advice. First of all, what I want to say is that non-coding RNA has a very large application prospect. This also means that it can be studied by the method of biosynthesis in the research of glioma. Similarly, the research of biosynthesis is only the initial exploratory research, so if you need to strengthen the reliability, it must be combined with other methods to increase its credibility. The fundamental experiment cycle is too long, and the current popular computer models are just in line. Just like the 4 articles mentioned by the reviewer, after reading carefully, the author found that these articles mainly describe a professional computer model for MiRNA–Disease Association prediction (IMCMDA). This is a surprising discovery, if the big data of Bioinformatics analysis is combined with this model, it can greatly improve the prediction of miRNAs for diseases, not only for gliomas, but for any other tumors, or even any other diseases. This will be an innovation in the era of computer big data, and the author will further explore the correlation between the two. At the same time, we have quoted these 4 articles on Model for MiRNA–Disease Association prediction (IMCMDA) into this article, and have discussed them in the discussion part of this research, in order to make this combined method familiar and understood by more people. Many thanks go to reviewers, and we are feel so warm for your suggestions.
