# Supplementary material for: Computational screening of potential glioma-related genes and drugs based on analysis of GEO dataset and text mining
Source: PLoS One. 2021 Feb 26;16(2):e0247612. doi: 10.1371/journal.pone.0247612 (PMC7909668; doi:10.1371/journal.pone.0247612)
Supplement: Attachment — Submitted filename: Response to Reviewers.docx [file pone.0247612.s002.docx]

Responds to the reviewers' comments:

Reviewer #1：

1. The authors have addressed or explained most of my previous points except Major point 1. Without supporting evidence from another gene expression dataset, the technical quality requirement, which is emphasized by PLoS One journal, could not be met. On the other hand, this is not a hard task: The authors can simply find another glioma RNA-Seq dataset from GEO, intersect its differentially expressed genes with GenCLIP3 gene set, and perform GO and KEGG functional enrichment analysis. If there are some overlap of the enriched function terms between the new and the previous analyses, the result of this manuscript should be much more consolidated.

Besides, I would also like to point out that adjusted p-value and FDR are actually NOT the same thing. There are several statistical methods for p-value adjustment against multiple tests; and FDR, often following the Benjamini family of methods, is one category of adjusted p-value.

Answer: We would like to thank the reviewer fort his comment. As reviewers have pointed out, without supporting evidence from another gene expression dataset, the technical quality requirement could not be met. In response to this, we followed the reviewer’s recommendations and methods, after continuous mining and exploration of other datasets in GEO, repeated analysis and verification, and finally found a dataset GSE109857. Through verification, some overlap of the enriched function terms between the new and the previous analyses, the final results have been listed and modified in the article.

Secondly, follow the reviewer’s description of both the FDR and the adjusted P-value, we have repeatedly checked the literature and found that, as the reviewer said, the two are not the same thing. Thank the reviewers for such important and valuable comments. And the threshold used in this article is FDR.

Reviewer #2：

1. Authors should carefully check the information of references. For example, the author of [64] should be Chen X, Wang L, Qu J, Guan N-N, Li J-Q and its volume, issue and page number should be 34(24): 4256-4265; the year, volume, issue and page number of [66] should be 2017 18(4):558-576; the author of [67] should be Chen X, Yin J, Qu J, Huang L and its page number should be e1006418.

Answer: We appreciate the reviewer for pointing out this fact. We have checked the references thoroughly are now all in a uniform format in the revised manuscript.
